# Overexpression of Thiamin Biosynthesis Genes in Rice Increases Leaf and Unpolished Grain Thiamin Content But Not Resistance to *Xanthomonas oryzae* pv. *oryzae*

**DOI:** 10.3389/fpls.2016.00616

**Published:** 2016-05-10

**Authors:** Wei Dong, Nicholas Thomas, Pamela C. Ronald, Aymeric Goyer

**Affiliations:** ^1^Department of Botany and Plant Pathology, Oregon State UniversityCorvallis, OR, USA; ^2^Hermiston Agricultural Research and Extension Center, Oregon State UniversityHermiston, OR, USA; ^3^Department of Plant Pathology, University of California, DavisDavis, CA, USA

**Keywords:** thiamin, engineering, rice, plants, vitamin B1, disease

## Abstract

Thiamin diphosphate (ThDP), also known as vitamin B1, serves as an enzymatic cofactor in glucose metabolism, the Krebs cycle, and branched-chain amino acid biosynthesis in all living organisms. Unlike plants and microorganisms, humans are not able to synthesize ThDP *de novo* and must obtain it from their diet. Staple crops such as rice are poor sources of thiamin. Hence, populations that mainly consume rice commonly suffer thiamin deficiency. In addition to thiamin’s nutritional function, studies in rice have shown that some thiamin biosynthesis genes are involved in resistance to *Xanthomonas oryzae*, which causes a serious disease in rice fields. This study shows that overexpression of two thiamin biosynthesis genes, 4-methyl-5-β-hydroxyethylthiazole phosphate synthase and 4-amino-2-methyl-5-hydroxymethylpyrimidine phosphate synthase, involved in the first steps of the thiazole and pyrimidine synthesis branches, respectively, increased thiamin content up to fivefold in unpolished seeds that retain the bran. However, thiamin levels in polished seeds with removed bran were similar to those found in polished control seeds. Plants with higher accumulation of thiamin did not show enhanced resistance to *X. oryzae*. These results indicate that stacking of two traits can enhance thiamin accumulation in rice unpolished grain. We discuss potential roadblocks that prevent thiamin accumulation in the endosperm.

## Introduction

Rice, *Oryza sativa* (Poaceae family), is the most important food crop of the developing world and the staple food of more than 3 billion people. Rice provides more than half of the calories for 520 million people in Asia. Rice was cultivated in 124 countries in 2013, with corresponding worldwide production of 745 million tons (FAOSTAT^1^). Asia, where about 90% of rice is grown, has more than 200 million rice farms (Source: International Rice Research Institute). Rice-based farming is the main economic activity for hundreds of millions of rural poor in this region. In Africa, rice is the fastest growing staple, and demand for rice has increased in Latin America and Caribbean countries. It is estimated that rice production will need to increase by more than 40% by 2030 to meet the projected demand.

Thiamin, in its diphosphate form (ThDP), serves as an enzymatic cofactor for several thiamin-dependent enzymes involved in glucose metabolism, the Krebs cycle, and branched-chain amino acid biosynthesis (Goyer, 2010; Rapala-Kozik, 2011). Severe thiamin deficiency leads to the lethal disease beriberi (Rindi, 1996; Harper, 2006; Lonsdale, 2006). Therefore, thiamin is an essential micronutrient for humans. Plants are the main dietary source of thiamin. Yet, major staple crops such as rice, corn, and wheat contain low levels of thiamin. Grain milling technologies remove the bran from intact brown rice grain to produce polished, white rice (Miller et al., 2002; Sun et al., 2010). Consumers prefer the taste, appearance, and digestibility of polished white rice, which consists primarily of starchy endosperm (Prakash et al., 2014; Singh et al., 2015). Because about 65–85% of vitamins, minerals, and micronutrients are located in the bran and embryo (Miller et al., 2002), a large part of the thiamin content is lost during the polishing process. As a result, cooked polished white rice contains only about 0.039 mg of thiamin per 195 g (about one cup) and provides only 3% of the Recommended Daily Allowance (RDA) compared to unpolished rice that contains 0.2 mg of thiamin per 195 g, providing 16% of the RDA (i.e., 1.2 mg/day for a healthy adult; USDA Nutrient Database for Standard References). Populations whose diets are largely based on polished white rice often suffer from thiamin deficiency (Rindi, 1996; WHO, 1999; Lonsdale, 2006). Fortification of white rice and other cereals products with thiamin has been implemented in several industrialized countries to prevent thiamin deficiency-related diseases (Backstrand, 2002), but these strategies are expensive and have not yet been adopted in developing countries where rice constitutes an important part of the diet. Biofortification of rice by genetic engineering or breeding offers an alternative approach that is cost-effective and sustainable (Mayer et al., 2008; Pourcel et al., 2013). Thus, strategies that boost the amount of thiamin in rice grain, particularly in the white starchy endosperm, would benefit populations that are not likely to adopt brown rice.

**FIGURE 1:**
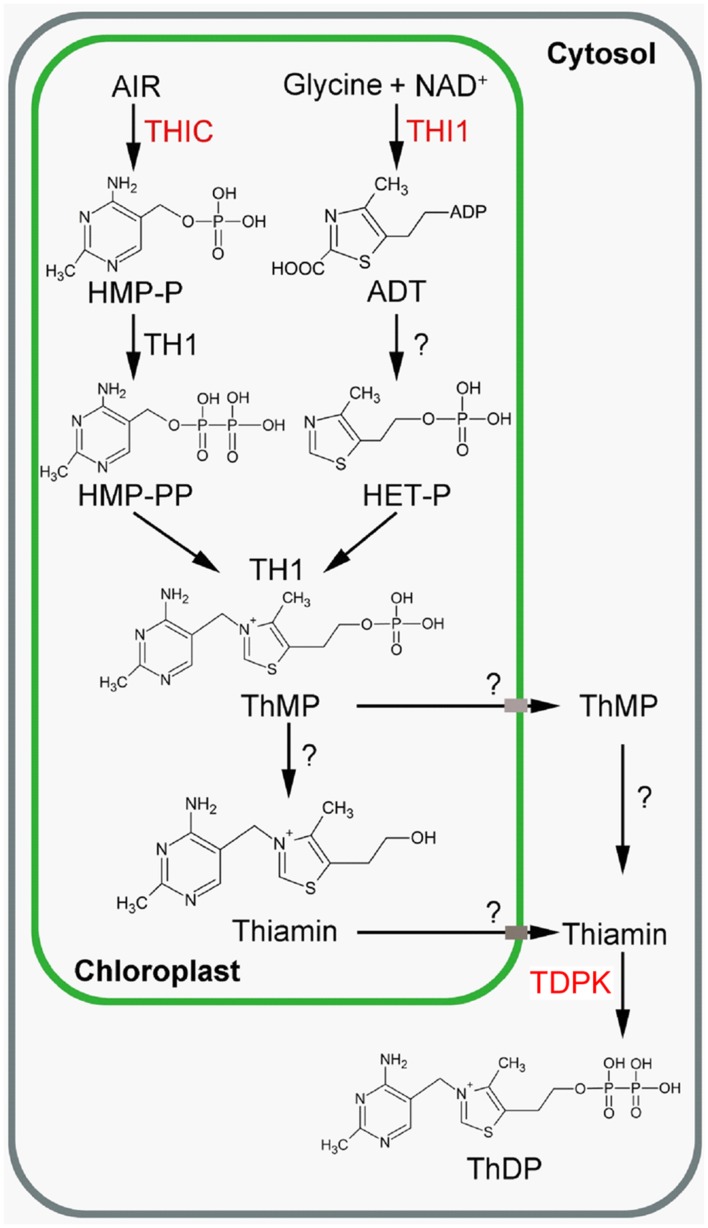
**Thiamin biosynthesis in plants (Source: Dong et al., 2015)**. Engineered enzymes are in red. ADT, adenosine diphospho-5-(β-ethyl)-4-methylthiazole-2-carboxylic acid; AIR, 5-aminoimidazole ribonucleotide; HET-P, 4-methyl-5-β-hydroxyethylthiazole phosphate; HMP-P, 4-amino-2-methyl-5-hydroxymethylpyrimidine monophosphate; HMP-PP, 4-amino-2-methyl-5-hydroxymethylpyrimidine diphosphate; NAD, nicotinamide adenine dinucleotide; ThMP, thiamin monophosphate; ThDP, thiamin diphosphate; THI1, HET-P synthase, THIC; HMP-P synthase; TDPK, Thiamin diphosphokinase; TH1, HMP-P kinase/ThMP pyrophosphorylase. The question mark indicates that the gene/enzyme has not been characterized yet.

Bacterial leaf blight which is caused by *X. oryzae* pv. *oryzae* is one of the most devastating diseases in major rice production areas in tropical Asia (Nino-Liu et al., 2006; Rajarajeswari and Muralidharan, 2006; Delteil et al., 2010). Two thiamin biosynthesis genes were reported to be involved in resistance to this pathogen. First, transgenic plants with reduced expression of *THI1*, which encodes HET-P synthase (**Figure 1**), were more susceptible to *X. oryzae* pv. *oryzae* (Wen et al., 2003; Wang et al., 2006). Second, a knockdown of *TDPK1*, which encodes the last enzyme in ThDP biosynthesis (**Figure 1**), in rice expressing the *XA21* resistance gene is more susceptible to *X. oryzae* pv. *oryzae* (Lee et al., 2011). It has also been shown that exogenous application of thiamin leads to enhanced resistance to *X. oryzae* pv. *oryzae* (Ahn et al., 2005).

In plants, *de novo* thiamin synthesis predominantly occurs in photosynthetic tissues (Colinas and Fitzpatrick, 2015). Thiamin is made of a thiazole and pyrimidine moieties. Synthesis of the thiazole and pyrimidine moieties, as well as fusion of the moieties to form thiamin monophosphate (ThMP), occur in chloroplasts (**Figure 1**). The first committed steps of thiazole and pyrimidine synthesis are catalyzed by 4-methyl-5-β-hydroxyethylthiazole phosphate (HET-P) synthase (THI1) and 4-amino-2-methyl-5-hydroxymethylpyrimidine phosphate (HMP-P) synthase (THIC), respectively (Belanger et al., 1995; Ribeiro et al., 1996; Raschke et al., 2007; Kong et al., 2008; Goyer, 2010). THI1 catalyzes the formation of an adenylated thiazole product (ADT, adenosine diphospho-5-(β-ethyl)-4-methylthiazole-2-carboxylic acid) from NAD, glycine and a sulfide group (Chatterjee et al., 2007) which is subsequently hydrolyzed to HET-P by a so-far-uncharacterized enzyme. In THI4, the homolog of THI1 in yeast, the sulfide is transferred from a conserved cysteine residue of THI4 (Chatterjee et al., 2011). THIC catalyzes the formation of HMP-P from 5-aminoimidazole ribonucleotide (AIR). THIC contains an [Fe–S] cluster that reduces *S*-adenosylmethionine (SAM) to give an adenosyl radical (Raschke et al., 2007; Chatterjee et al., 2008). This radical participates in the isomerization of AIR to HMP-P. HMP-P is then phosphorylated to HMP-PP and condensed to HET-P by a bifunctional HMP-P kinase/ThMP pyrophosphorylase (TH1) to form ThMP (Kim et al., 1998; Ajjawi et al., 2007b; Rapala-Kozik et al., 2007). ThMP is then dephosphorylated to thiamin by at least one phosphatase from the HAD phosphatase family (ThMPase; Hasnain et al., 2016). Thiamin is then pyrophosphorylated to ThDP by ThDP kinase (TDPK) in the cytosol (Ajjawi et al., 2007a).

Engineering of thiamin biosynthesis was recently attempted in *Arabidopsis* (Dong et al., 2015) by overexpressing cDNAs encoding THI1 and THIC. While single-gene *THI1* or *THIC* plants had similar thiamin levels to those of the wild-type, simultaneous overexpression of both *THI1* and *THIC* increased thiamin levels up to 3.4- and 2.6-fold in leaves and seeds, respectively. Thiamin-accumulating plants also limit populations of *Pseudomonas syringae* after mechanical inoculation (Dong et al., 2015).

In this paper, we report the effect of overexpressing rice cDNAs encoding THI1, THIC, and TDPK1 under the control of the constitutive maize ubiquitin promoter. We report the effects of overexpressing these genes on leaf and seed thiamin levels and on resistance to *X. oryzae* pv. *oryzae* in Kitaake rice. While single-gene, overexpressing plants did not accumulate thiamin, plants that contain both *THI1* and *THIC* overexpression loci had up to fivefold increased thiamin content in unpolished seed compared to the wild type. Thiamin-accumulating plants did not display altered resistance to *X. oryzae* pv. *oryzae*.

## Materials and Methods

### Plant Expression Vector Construction

The coding sequences of *THIC* and *THI1* genes were amplified by PCR from a Nipponbare rice leaf cDNA library using Phusion DNA polymerase (New England Biolabs, Ipswich, MA, USA) or PrimeStar polymerase (Clontech, Mountain View, CA, USA) using the primers shown in Supplementary Table 1. Amplicons were cloned into pENTR-D-TOPO vector to make pENTR-D-TOPO-OsTHIC and pENTR-D-TOPO-OsTHI1 vectors, and constructs were verified by sequencing. A 2.2-kb cassette containing *THIC* and *att*L recombination sites was amplified by PCR from pENTR-D-TOPO-THIC using primers M13Fwd and M13Rev, and cloned into the binary vector UbiNC1300RFCA using LR clonase (Invitrogen, Carlsbad, CA, USA). The UbiNC1300RFCA vector is a modified version of Ubi-C1300 (Chern et al., 2005) in which the Reading Frame Cassette A containing *att*R sequences, *ccd*B gene, and chloramphenicol-selection gene was ligated in *Sma*I site. The backbone of the UbiNC1300RFCA vector is pCAMBIA1300 which contains the *hpt*II gene for selection on hygromycin. The pENTR-D-TOPO-OsTHI1 vector was linearized with *Eco*RI and the digestion product was used in LR clonase reaction to subclone *OsTHI1* into UbiNC1300RFCA or UbiNC4300RFCA. The UbiNC4300RFCA vector is identical to UbiNC1300RFCA but contains the phosphomannose isomerase (*PMI*) gene in place of *hpt*II for selection on medium supplemented with mannose (Lucca et al., 2001). Each coding sequence was under the control of the strong constitutive maize *ubiquitin* gene promoter with its non-translated first exon and intron (Christensen and Quail, 1996). A construct containing the *TDPK1* cDNA under the control of the *ubiquitin* gene promoter was previously made (Lee et al., 2011). The resulting DNA constructs were introduced into *Agrobacterium tumefaciens* strain EHA105.

### Production of Genetically Engineered Rice Plants

DNA constructs were introduced into the Japonica variety Kitaake by *Agrobacterium*-mediated transformation as described previously (Hiei et al., 1994; Chern et al., 2005). TDPK1 and THIC overexpression lines were regenerated and selected on medium containing hygromycin. THI1 lines were regenerated and selected on medium containing either hygromycin or mannose (Lucca et al., 2001). The transgenic plants were screened for *THI1*, *THIC*, and *TDPK1* constructs by PCR with a primer located in the *ubiquitin* promoter (Ubi promoter F) and gene specific primers from their coding regions (Thi1_C4300_AS for *THI1*, ThiC_C1300_AS for *THIC*, and TPK(N2)_C1300_AS for *TDPK1*), respectively (Supplementary Table 1 and Supplementary Figures 1A,B).

Confirmed T_2_ progenies from lines THI1-7-31 and THIC-1-30 (Supplementary Figure 1D) were used for crosses to generate THI1 × THIC overexpressing lines. These lines were used because their flowering times coincided. THIC-1-30 T_2_ progeny all tested positive for the *THIC* transgene (*n* = 9). Eight out of 9 THI1-7-31 T_2_ progeny tested positive for the *THI1* transgene. Both THI1-7-31 and THIC-1-30 progenies (plants 97, 99, 100, 101, 133, 134, 135, 136) were used as donors (male) for reciprocal crosses (Supplementary Figure 1D). Pollen from plants 97, 99, 100, and 101 on one hand, and plants 133, 134, 135, and 136 on the other hand, were pooled and used as donor. F_2_ progeny from F_1_ lines 1, 5, 8, 25, and 31 were used for further experiments. Transgenes were determined using transgene specific primers (Supplementary Table 1).

### RNA Isolation and Real-Time Quantitative RT-PCR

Leaf samples (∼100 mg) were collected and immediately frozen in liquid nitrogen. Tissues were homogenized in Tissue Lyser (17s *s*^-1^, 1.5 min; Qiagen, Hilden, Germany). TRIZOL Reagent (1 mL; Invitrogen, Carlsbad, CA, USA) was added and vortexed for 5–10 min. Chloroform (200 μL) was added and the mixture was vortexed for 5 min. After centrifuging at 12 000 rpm for 15 min at 4°C, the aqueous phase was transferred to a new tube containing isopropanol to precipitate RNA. Samples were treated with DNase I to eliminate any trace of DNA according to the manufacturer’s recommendations (Roche, Basel, Switzerland). NucleoSpin RNA column II (Macherey-Nagel, Dueren, Germany) was used for RNA clean up. After RNA quantification using a nano drop ND-1000 Spectrophotometer (NanoDrop Technologies Inc., Wilmington, DE, USA), RNAs (1 μg) were reverse-transcribed to cDNAs with the AB high-capacity cDNA reverse transcription kits (Applied Biosystems, Foster City, CA, USA). cDNAs were diluted four times and 1 μL of cDNAs was used as template in 20-μL PCR reactions containing the SsoFast EvaGreen Supermix (Bio-Rad, Hercules, CA, USA) and 500 nM of forward and reverse primers. PCR reactions were performed on a Bio-Rad CFX 96 Real-Time System. PCR conditions were: (95°C for 3 s and 60°C for 3 s) 40 cycles followed by melt curve analysis. The housekeeping gene *ubiquitin* (*Os03g0234200*) was used as control for QPCR analysis. Primers sequences are listed in Supplementary Table 1. Primers efficiency was determined using the protocol described before (Schmittgen and Livak, 2008). Relative gene expression was calculated by using the 2^-ΔΔ^*^Ct^* method (Schmittgen and Livak, 2008).

### Thiamin Determination

Thiamin was analyzed by HPLC as described before (Dong et al., 2015). Thiamin and its phosphate esters were extracted from ∼100 mg or ∼20 mg of leaf or seed tissue, respectively, in 4 ml of 0.1 N HCl. Samples were sonicated in a water bath for 30 min, then centrifuged at 14 000 rpm for 10 min. Samples of 300 μL of the supernatant were mixed with 50 μL of freshly made 10 mM K_4_Fe(CN)_6_, which was dissolved in 3.7 N NaOH, and 100 μL of HPLC-grade methanol. The samples were vigorously shaken, sonicated for 5 min, and centrifuged at 14 000 rpm for 10 min. Thiamin, ThMP, and ThDP were separated on a Capcell Pak NH_2_ column (5 mm, 4.6 × 150 mm i.d.; Shiseido, Tokyo, Japan) using a 4:6 (v/v) solution of 90 mM potassium phosphate buffer, pH 8.4, and acetonitrile as mobile phase. The analyses were performed using a UltiMate 3000 HPLC system (Thermo Fisher Scientific, Waltham, MA, USA) equipped with a WPS-3000TSL autosampler, a TCC-3000 column compartment set at 25°C, an LPG-3400SD quaternary analytical pump, and an FLD-3000 fluorescence detector. Chromatograms were integrated using the Chromeleon^TM^ 7.1 chromatography data system (Dionex, Sunnyvale, CA, USA). The flow rate was 0.5 mL/min, and the volume injected was 5 μl. Thiochrome derivatives were detected by fluorescence at excitation 365 nm and emission 435 nm. Detector response was calibrated by using thiamin, ThMP, and ThDP standards.

### *Xanthomonas oryzae* pv. *oryzae* Resistance

*Xanthomonas oryzae* pv. *oryze* strain PXO99A was used to infect rice plants (Salzberg et al., 2008). *X. oryzae* pv. *oryzae* was grown on peptone sucrose agar plates for 3 days and resuspended in water for an OD_600_ of ∼0.5. Five-week old greenhouse grown plants were transferred to controlled growth chambers set to 28°C, 80% relative humidity, and 14 h light and 10 h dark cycling. Plants were left to acclimate for 2–3 days before inoculations. Plants were inoculated by cutting leaf tips with scissors dipped in *X. oryzae* pv. *oryzae* suspension media (Kauffman et al., 1973). Lesion measurements were scored 14 days after scissor inoculation.

## Results

### Production of Rice Plants Containing THI1, THIC, or TDPK1 Transgenes

**FIGURE 2:**
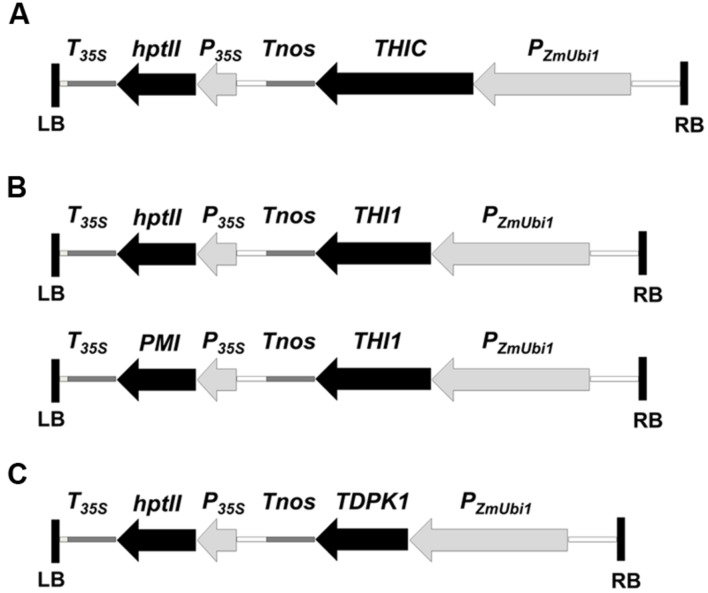
**Plant transformation vectors for the overexpression of *THIC***(A)**, *THI1***(B)**, and *TDPK1***(C)****. The coding sequences of *THIC*, *THI1*, and *TDPK1* were cloned downstream of the maize *ubiquitin-1* promoter (*P_ZmUbi1_*) and upstream of the nopaline synthase terminator (*T_nos_*). *THIC* and *TDPK1* were cloned in the UbiNC1300RFCA and Ubi-C1300 vectors, respectively. Both vectors are derived from the pCAMBIA1300 vector which carries the hygromycin phosphotransferase (*hpt*II) gene as a selectable marker. *THI1* was cloned in the UbiNC1300RFCA and the UbiNC4300RFCA. The UbiNC4300RFCA vector is derived from UbiNC1300RFCA and carries the phosphomannose isomerase gene (*PMI*) as a selectable marker. *P_zmUbi1_*, the maize *ubiquitin-1* promoter with its non-translated first exon and intron; *T_nos_*, nopaline synthase terminator; *P_35S_*, cauliflower mosaic virus 35S promoter; *T_35S_*, cauliflower mosaic virus 35S terminator; *hpt*II, hygromycin phosphotransferase gene; *PMI*, phosphomannose isomerase gene; LB and RB, left and right T-DNA borders, respectively.

The coding sequences of the thiamin biosynthesis genes *THIC* and *THI1* were amplified by PCR from a Nipponbare rice leaf cDNA library. The amplified *THIC* sequence was cloned into the plant transformation binary vector UbiNC1300RFCA, which contains the *hpt*II gene for selection on hygromycin. The amplified *THI1* sequence was first cloned in the vector UbiNC4300RFCA, which contains the *PMI* gene for selection on medium supplemented with mannose (Lucca et al., 2001). Because we were able to regenerate only one plant carrying the transgene using mannose selection (plant THI1-12 thereafter), we re-cloned *THI1* into UbiNC1300RFCA. Both *THIC* and *THI1* were under the control of the ubiquitin promoter (**Figure 2**). The *TDPK1* cDNA was isolated as described and introduced into the Ubi-C1300 binary vector under the control of the ubiquitin promoter (Chern et al., 2005; Lee et al., 2011). The DNA constructs were introduced into the Kitaake cultivar by *Agrobacterium*-mediated transformation of rice callus as described (Chern et al., 2001). T_0_ THIC, THI1, and TDPK1 plants were regenerated on selection medium containing hygromycin or mannose. The presence of the constructs was verified by PCR genotyping (Supplementary Figure 1A). In total, we obtained 11 THI1, 10 THIC, and 24 TDPK1 independently transformed lines.

### Characterization of THI1, THIC, and TDPK1 Transgenic Lines

**FIGURE 3:**
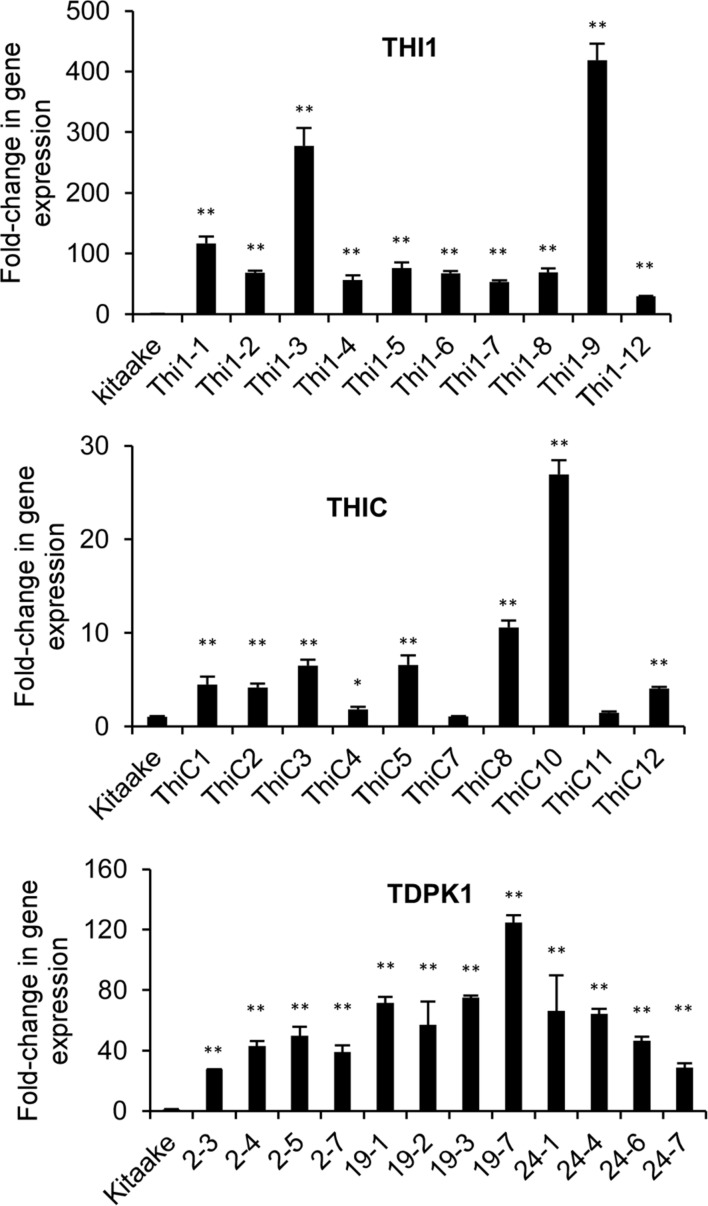
**Quantification of *THI1*, *THIC*, and *TDPK1* gene expression in leaf of transgenic THI1, THIC, and TDPK1 plants by real-time quantitative RTPCR**. T_0_ generations were used for *THI1* and *THIC*, T_1_ generation was used for *TDPK1*. Each line used was positive by PCR genotyping (Supplementary Figures 1A–C). The housekeeping gene *ubiquitin* (*Os03g0234200*) was used for normalization. Relative gene expression was calculated by using the 2^-ΔΔ^*^Ct^* method. Note that the Thi1-10 line died and could not be analyzed. Data are means ± SE of three determinations. Asterisks indicate significant difference compared to the Kitaake control as determined by Student *t*-test (^∗^*p* < 0.05; ^∗∗^*p* < 0.01).

We used real time quantitative RT-PCR to measure the expression of *THI1*, *THIC*, and *TDPK1* in leaf samples of T_0_ or T_1_ plants. The housekeeping gene *ubiquitin* (*Os03g0234200*) was used as control. *THI1* gene expression in THI1 lines increased between 50 and 400 times compared with the Kitaake control, Lines Thi1-3 and Thi1-9 accumulated the highest amount of *THI1* transcripts (**Figure 3**). *THIC* gene expression in THIC lines increased between 1.5 and 27 times compared with the Kitaake control, with lines THIC-8 and THIC-10 accumulating the highest amount of *THIC* transcripts (**Figure 3**). *TDPK1* gene expression in T_1_ progeny of T_0_ *TDPK1* lines # 2, 19, and 24 increased between 20 and 120 times compared to the Kitaake control (**Figure 3**).

**FIGURE 4:**
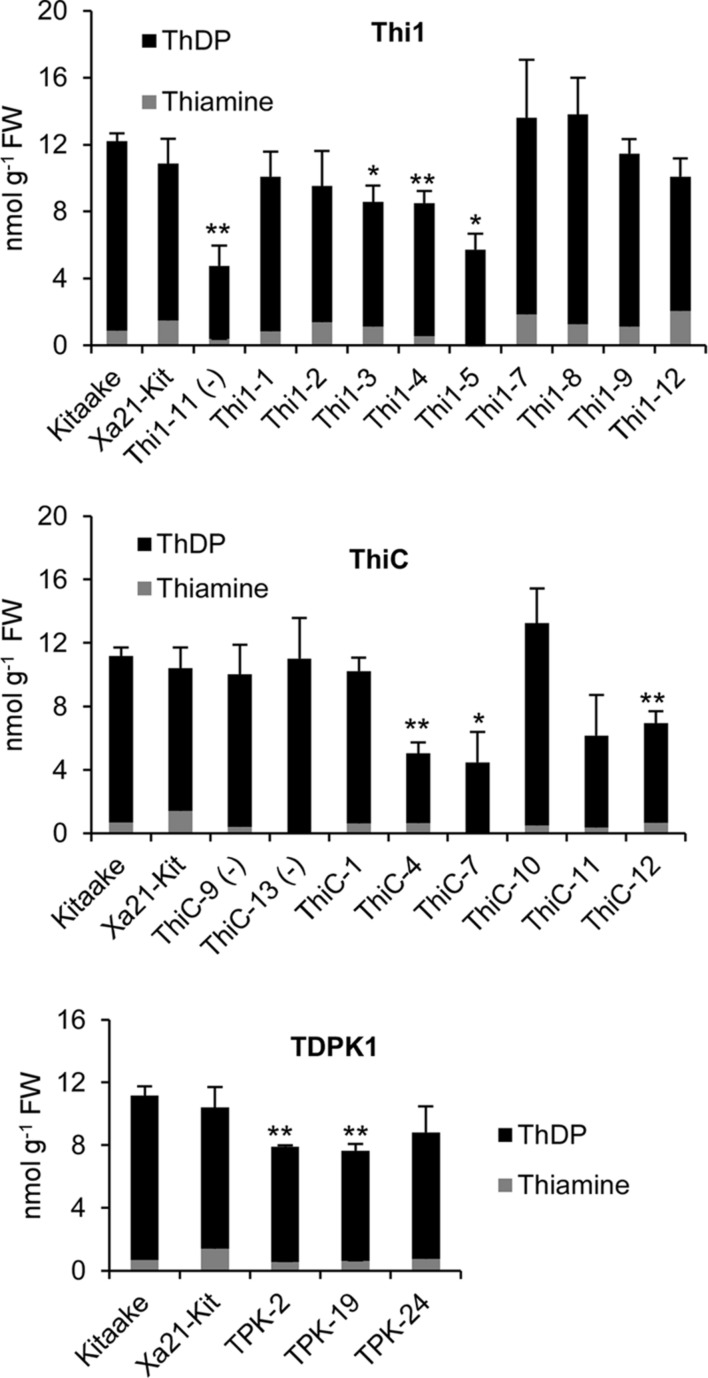
**Thiamin and ThDP concentrations in leaves of T_1_ THI1, THIC, and TDPK1 lines**. Each individual plant was PCR genotyped for the presence of the corresponding gene. Negative signs in parenthesis indicate rice lines that were genotyped negative. Wild-type Kitaake was used as a control. ThMP could not be detected in any sample. Xa21-Kit, Kitaake expressing *XA21*, a gene that confers strong resistance to *Xanthomonas oryzae* pv. *oryzae*. Data are means ± SE of at least three determinations. Asterisks indicate significant difference compared to the Kitaake control as determined by Student *t*-test (^∗^*p* < 0.05; ^∗∗^*p* < 0.01).

Then, we determined levels of thiamin (i.e., free thiamin, ThMP, and ThDP) in leaves of T_1_ *THI1*, *THIC*, and *TDPK1* plants carrying the corresponding transgene (**Figure 4**). None of THI1, THIC, or TDPK1 plants accumulated thiamin in higher amounts than the Kitaake control (*p* < 0.05).

Finally, we tested T_1_ THI1, THIC, and T_0_ and T_1_ TDPK1 lines for resistance against *X. oryzae* pv. *oryzae* (Supplementary Figures 2A–D). Each plant was tested for the presence of the transgene (Supplementary Figures 1A–C). Wild-type Kitaake was used as negative control (i.e., susceptible), and Kitaake expressing *XA21*, a gene that confers strong resistance to *X. oryzae* pv. *oryzae*, was used as positive control (i.e., resistant). As shown in Supplementary Figure 2, lesion lengths ranged between 12 and 20 cm in Kitaake, and between 0.75 and 2.5 cm in *XA21*-expressing plants. Lesion lengths in THI1 and THIC lines were not significantly different than those in Kitaake and/or negative segregants. Individuals segregating for each transgene and null segregants showed similar disease phenotypes comparable to the susceptible Kitaake parent line. Lines TPK-2, TPK-19, and TPK-24 showed shorter lesions than Kitaake, with significant difference in the case of TPK-19, suggesting enhanced resistance to *X. oryzae* pv. *oryzae*. To validate this observation, we planted 10 to 12 T_1_ segregating seeds for each of these lines, identified positive segregants carrying the transgene by PCR (Supplementary Figure 1C), and evaluated them for resistance to *X. oryzae* pv. *oryzae* (Supplementary Figure 2D). This analysis indicated no significant lesion length difference (*p* < 0.05) between T_1_ TDPK1 plants and Kitaake control plants. Given the lack of thiamin accumulation in THI1, THIC, and TDPK1 plants, these results are not surprising. We hypothesized that overexpression of THI1 and THIC together, and possibly TDPK1 as well, would lead to higher levels of thiamin accumulation.

### Thiamin Levels in THI1 × THIC Lines

**FIGURE 5:**
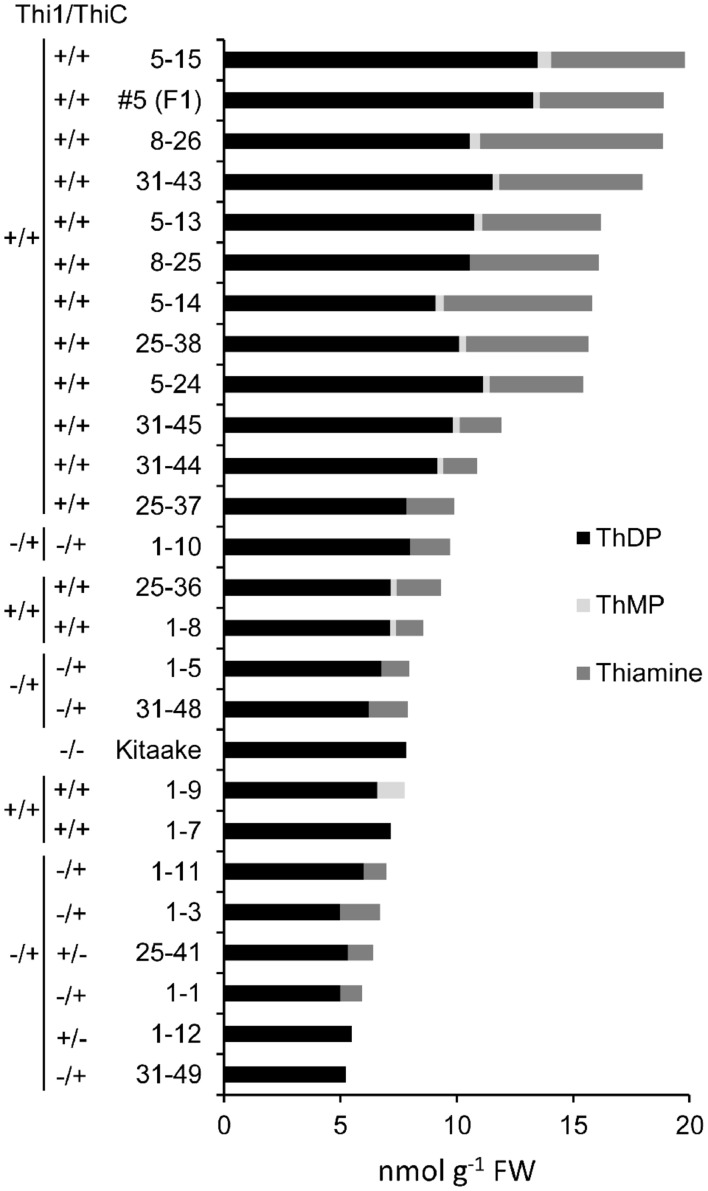
**Thiamin, ThMP, and ThDP concentrations in leaves of F_2_ progeny from crosses between THI1 and THIC lines**. T_2_ progenies from lines THI1-7-31 and THIC-1-30 were used in reciprocal crosses to generate THI1 × THIC overexpressing lines. These lines were used because their flowering times coincided. F_2_ progenies from F_1_ lines 1, 5, 8, 25, and 31 were used for analysis. Each F_2_ individual plant was tested for the presence (+) or absence (–) of the *THI1* and *THIC* transgenes as indicated and also shown in Supplementary Figure 1F. #5 (F1) is the F_1_ parent of F_2_ progenies 5–13, 5–14, 5–15, and 5–24 and was included in the analyses. Data are means of two determinations.

**FIGURE 6:**
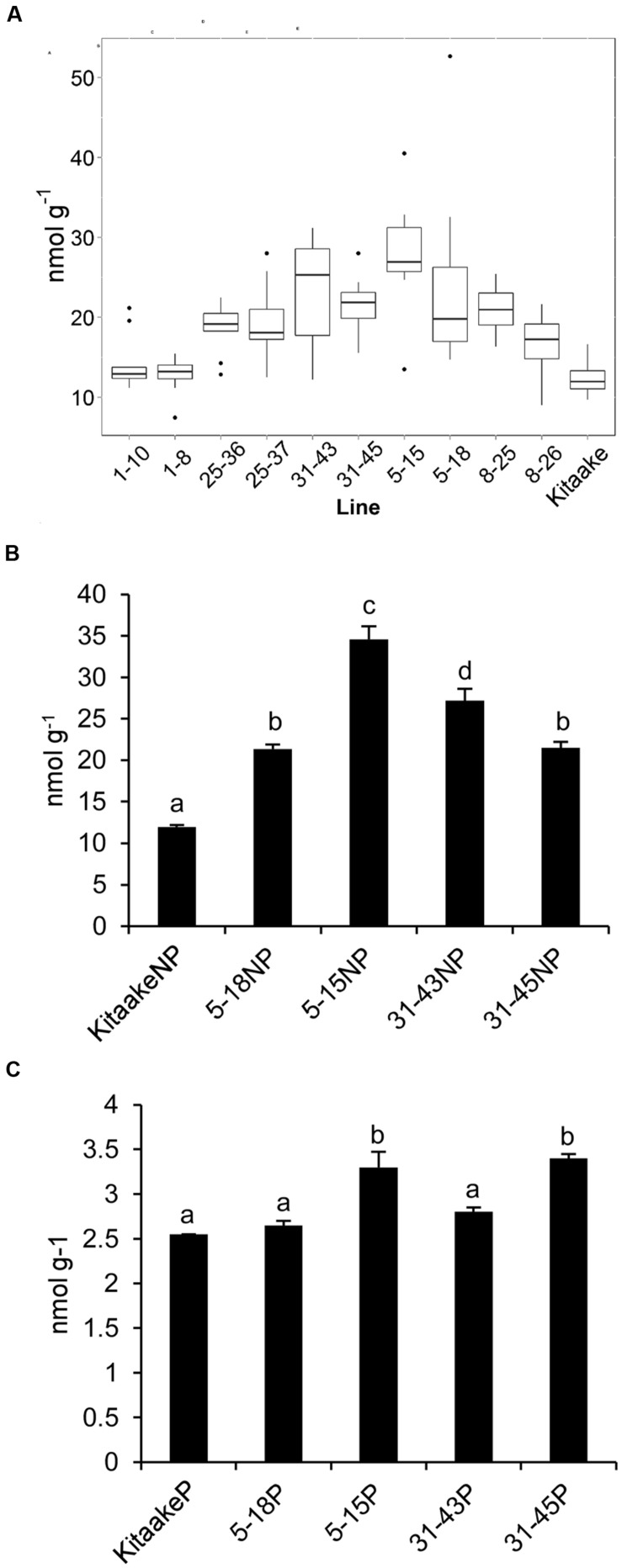
**Thiamin concentrations in F_3_ seeds of THI1 × THIC lines**. **(A)** Boxplot representation of thiamin concentrations in 10 to 12 individual unpolished seeds of several independent THI1 × THIC lines. **(B)** Thiamin concentrations in unpolished (NP) seeds of Kitaake and several independent THI1 × THIC lines. Twenty unpolished seeds per genotype were pooled and reduced to powder for thiamin analysis. Data are means ± SE of three determinations. **(C)** Thiamin concentrations in polished (P) seeds of Kitaake and several independent THI1 × THIC lines. Twenty seeds per genotype were polished, pooled, and reduced to powder for thiamin analysis. Data are means ± SE of three determinations. Identical letters indicate that there was no significant difference between samples as determined by ANOVA (*p* > 0.05).

**FIGURE 7:**
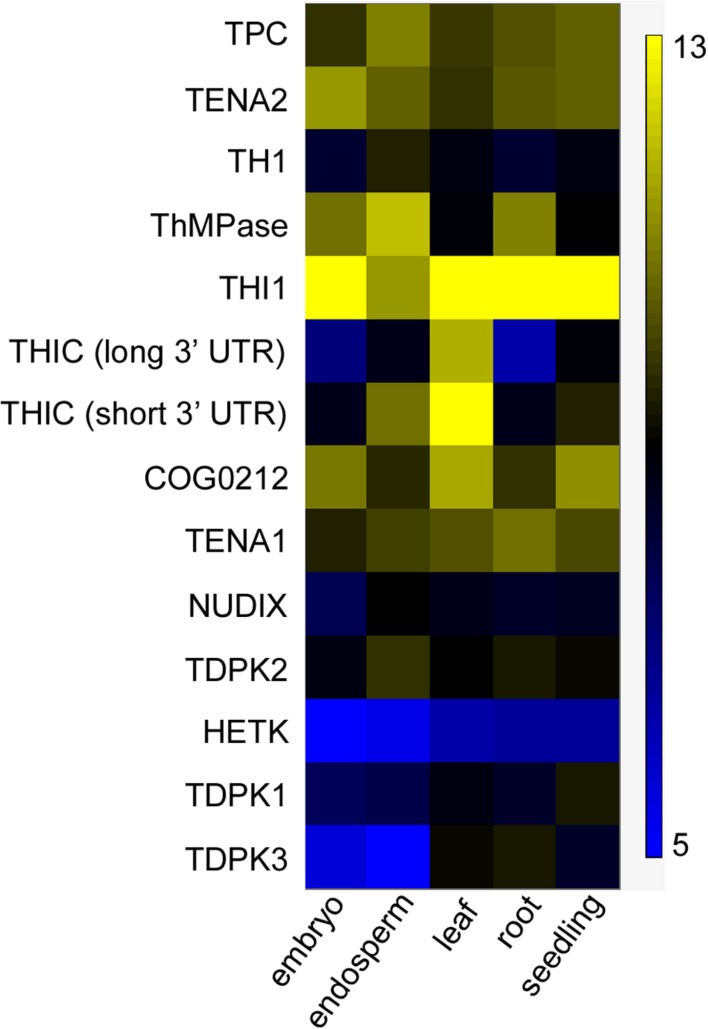
**Expression of thiamin metabolism genes in different organs in rice**. Data were computed from the Rice Oligo Array Database using the Affymetrix platform (http://ricearray.org/index.shtml). Heatmap scale indicates log2 intensity of fluorescence. Embryo and endosperm of seed were collected at 6 days after anthesis. Leaves and roots were from 7-day-old seedlings. Seedlings samples were from 10-day-old seedlings. All tissues were from *Oryza sativa* japonica cultivar Zhonghua 11 cultivated at day/night temperatures 28/22°C in a phytotron.

**FIGURE 8:**
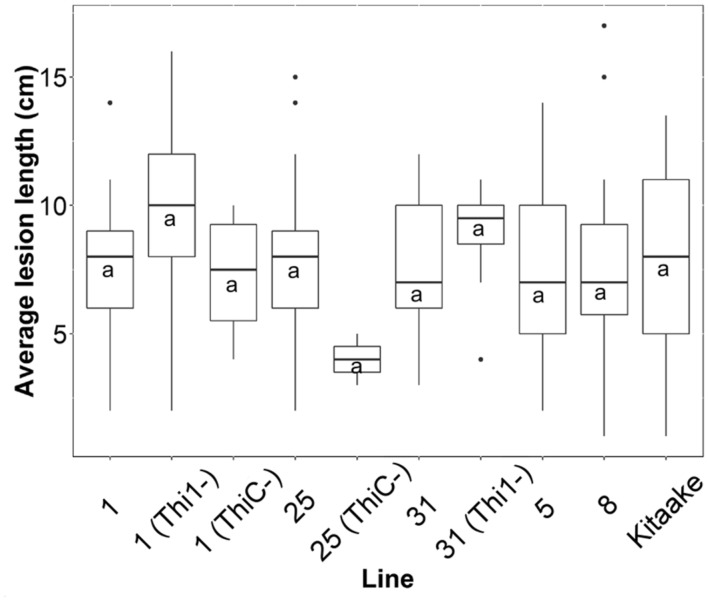
**Average lesion lengths from F_2_ progeny derived from five THI1 × THIC lines infected with *X. oryzae* pv. *oryzae***. Lesion lengths were measured 2 weeks after *X. oryzae* pv. *oryzae* inoculation (strain PXO99A). F_2_ progeny derived from five F_1_ lines were classified by PCR for the presence or absence of *THI1* and *THIC* transgenes (Supplementary Figure 1F). On the x-axis, ‘Kitaake’ represents 25 lesion measurements from six individuals. ‘1’ represents 21 lesion measurements from five F_2_ progeny derived from F_1_ line #1 that carry both *THI1* and *THIC*. ‘1(Thi1-)’ represents 25 measurements from six F_2_ progeny derived from F_1_ line #1 that only carry *THIC*. ‘1(ThiC-)’ represents four measurements from one F_2_ individual derived from F_1_ line #1 that only carries *THI1*. ‘25’ represents 34 measurements from eight F_2_ progeny derived from F_1_ line #25 that carry both *THI1* and *THIC*. ‘25(ThiC-)’ represents two measurements from one F_2_ individual derived from F_1_ line #25 that only carries *THI1*. ‘31’ represents 33 lesion measurements from seven progeny derived from F_1_ line #31 that carry both *THI1* and *THIC*. ‘31(Thi1-)’ represents eight measurements from two F_2_ progeny derived from F_1_ line #31 that only carry *THIC*. ‘5’ represents 49 lesion measurements from ten F_2_ progeny derived from F_1_ line #5 that carry both *THI1* and *THIC*. ‘8’ represents 20 lesion measurements from four progeny derived from F_1_ line #8 that carry both *THI1* and *THIC*. The letter “a” indicates statistical grouping determined by Tukey–Kramer HSD analysis (alpha = 0.05).

T_2_ individuals from T_1_ lines THI1-7-31 and THIC-1-30 carrying the *THI1* and *THIC* overexpression transgenes (Supplementary Figure 1D) were used in reciprocal crosses to produce THI1 × THIC overexpression lines. Five F_1_ lines (1, 5, 8, 25, and 31) carrying both transgenes as determined by PCR genotyping (Supplementary Figure 1E) were self-pollinated. F_2_ and F_3_ progenies were used for further analysis. PCR genotyping of 50 F_2_ progenies is shown in Supplementary Figure 1F. Twenty-five F_2_ progenies, including 16 that carry both *THI1* and *THIC* transgenes, were analyzed for thiamin. Total thiamin levels were up to 2.5-fold higher in F_2_ leaves containing both *THI1* and *THIC* transgenes (i.e., F_2_ progeny 5–15) than in Kitaake leaves (**Figure 5**). Ten to 12 individual F_3_ seeds per F_2_ progeny carrying both transgenes were then analyzed for thiamin content. Data are shown in a box and whisker plot in **Figure 6A**. For all the lines analyzed, except lines 1–10 [which carries only the *THIC* transgene (**Figure 5**)] and 1–8, the median and maximal values were higher than those in Kitaake (**Figure 6A**). Some seeds (e.g., F_3_ seeds from the F_2_ 5–15 and 5–18 progenies) contained fourfold to fivefold the amount of thiamin found in Kitaake seeds (shown as outliers on **Figure 6A**). We then compared thiamin content in pools of 20 seeds, either unpolished or polished, for each of the lines 5–18, 5–15, 31–43, and 31–45, and Kitaake. While thiamin levels were higher in unpolished F_3_ seeds than in unpolished Kitaake seeds (1.8- to 2.9-fold; **Figure 6B**), they were similar in polished grains, with a maximum increase of 1.3-fold (**Figure 6C**), showing that thiamin accumulated mainly in the bran of F_3_ seeds, and not in the endosperm.

### *In Silico* Expression Analysis of Thiamin Metabolism Genes

Because thiamin accumulated mostly in the bran of both wild-type and engineered Kitaake, we hypothesized that the rice endosperm does not express thiamin biosynthesis genes. Supporting this hypothesis, mostly photosynthetic tissues are active sites of *de novo* thiamin synthesis (Colinas and Fitzpatrick, 2015). To test our hypothesis, Affymetrix microarray dataset from the Rice Oligo Array Database^2^ was used to study expression of 13 known thiamin metabolism genes (Supplementary Table 2) in grain, leaf, root, and seedling tissues (**Figure 7**). *THI1* was expressed in all tissues, including embryo and endosperm, and was the most highly expressed gene amongst thiamin metabolism genes in all tissues analyzed. Two *THIC* transcripts were detected: one transcript with short 3′ untranslated region (UTR; type II) which drives high *THIC* expression, and one with long 3′ UTR (type III) which drives low *THIC* expression (Wachter et al., 2007). The short 3’ UTR *THIC* transcript was detected at higher level than the long 3’ UTR *THIC* transcript in all tissues. The short 3′ UTR *THIC* transcript was detected at the highest level in leaf tissue, an organ that produces high thiamin levels, and at relatively high level in the endosperm. Both *THIC* transcript variants as well as *TH1* transcripts were detected at low level in embryo and root, an organ which cannot produce thiamin at a sufficient rate for growth (Goyer, 2010). *TH1* transcripts were detected at the highest level in the endosperm amongst tissues. *ThMPase* transcripts were detected in relatively high amounts in embryo, endosperm, and roots, and at lower levels in leaves and seedlings. *TDPK1* and *TDPK3* transcripts were expressed at relatively low levels in the endosperm, which is consistent with free thiamin being the predominant form of thiamin found in seeds. *TDPK2* transcripts were expressed at slightly higher levels than *TDPK1* and *TDPK3* transcripts throughout tissues. In summary, these data indicate that the genes necessary for *de novo* thiamin biosynthesis, *THIC*, *THI1*, *TH1*, and *ThMPase* are all expressed in the endosperm at 6 days after anthesis. Although it will be important to analyze gene expression at the later stages of endosperm development, these results suggest that the low amounts of thiamin in the endosperm may be due to a lack of functional thiamin biosynthesis proteins and/or the absence of precursors in sufficient amounts rather than the absence of transcripts.

### *Xanthomonas oryzae* pv. *oryzae* Resistance in THI1 × THIC Lines

We inoculated 50 F_2_ progenies from F_1_ lines 1, 5, 8, 25, and 31, and the wild type Kitaake with *X. oryzae* pv. *oryzae*, and measured lesion lengths on at least two leaves per plant (*n* = 213). Each F_2_ progeny was genotyped for *THI1* and *THIC* transgenes (Supplementary Figure 1F). There was no statistical difference between mean lesion lengths of F_2_ individuals that carry both transgenes (*n* = 150) or only one of the transgenes (*n* = 38) and lesions from Kitaake controls (*n* = 25; *p* = 0.3; **Figure 8**). In addition, there was no statistically significant correlation (*p* > 0.05) between lesion lengths and thiamin, ThMP, ThDP, or total thiamin content determined in 24 F_2_ individuals (Supplementary Figure 3).

## Discussion

Thiamin biofortification of rice grain is an attractive approach to provide adequate levels of thiamin to the global population and contribute to the eradication of thiamin deficiency in the diet. In this study, a two-gene strategy based on the overexpression of *THI1* and *THIC* genes under the control of the constitutive *ubiquitin* promoter enhanced thiamin content up to fivefold in the unpolished, brown rice grain. Plants that only overexpressed *THI1* or *THIC* did not accumulate thiamin in any tissue measured in this study. Similar results were recently reported in *Arabidopsis*, with total thiamin fold-increase of 3.4 and 2.6 in leaves and seeds, respectively, of *THI1* ×*THIC* engineered plants (Dong et al., 2015), while single gene-overexpressing plants did not accumulate thiamin. Altogether, these results suggest that overexpression of both *THI1* and *THIC* is the minimal requirement for increased thiamin accumulation in plants.

The thiamin increase in whole grain of THI1 × THIC lines was lost in polished white rice grain. This suggests that the increase in thiamin content was mostly or entirely in the bran. Several scenarios could explain these results. First, the *THI1* and *THIC* transgenes may not be expressed in the endosperm. However, this possibility seems unlikely as the ubiquitin promoter, when fused to the β-glucuronidase gene, was shown to drive the expression of β-glucuronidase in the endosperm of rice grain (Takaiwa et al., 2007). Nevertheless, overexpressing *THI1* and *THIC* under the control of endosperm-specific promoters such as glutelin should be tested in future studies. Second, the endosperm may not be a *de novo* production site for thiamin. Instead, thiamin or its precursors may be transported to the endosperm from maternal sources. For instance, *Arabidopsis* seeds and maize kernels can acquire thiamin from maternal tissues (Shimamoto and Nelson, 1981; Goyer, 2010; Guan et al., 2014), and gene expression analysis indicates that the seed may produce thiamin by coupling *de novo* thiazole biosynthesis with pyrimidine salvage (Guan et al., 2014). However, rice microarray gene expression analysis shows that the genes necessary for *de novo* thiamin biosynthesis are all expressed in the endosperm at 6 days after anthesis. This suggests that the endosperm can produce thiamin at some point during seed development. It is possible that the endosperm loses its capability for *de novo* thiamin production later during seed development by switching off the expression of thiamin biosynthesis genes as was reported for *THIC* in maize endosperm (Guan et al., 2014). If this is the case, one can assume that synthesis of the precursors of thiamin’s thiazole and pyrimidine moieties, namely NAD, AIR, and glycine, also decreases during seed development. This lower availability of NAD, AIR, and glycine can limit thiamin production in the endosperm of rice seeds that express *THI1* and *THIC* transgenes. Another possible limiting factor is the requirement of THIC for other proteins to be active. THIC contains an iron–sulfur cluster which catalyzes the formation of the 5′-deoxyadenosyl radical from *S*-adenosylmethionine that is necessary for its activity (Raschke et al., 2007). The iron–sulfur cluster must be reduced for this reaction to occur. Affinity chromatography approaches identified THIC as a potential target of chloroplastic thioredoxins (Balmer et al., 2003), which suggests that THIC activity is dependent of the thioredoxin/ferredoxin system (Pourcel et al., 2013). This photosynthesis-dependent system may not be operative throughout endosperm development, thereby negating the effect of *THIC* overexpression. Therefore, it may be that overexpression does not correspond to elevated functional protein levels in the endosperm. Future analysis to assess protein accumulation is needed.

TDPK1 overexpression had no effect on total thiamin pools and on thiamin profiles. One possible explanation is that *TDPK1* transcripts accumulation does not correlate with TDPK1 protein accumulation. Future analysis to assess TDPK1 accumulation is needed. It will also be important to determine the subcellular localization of TDPK1 and its two homologs. The rice genome contains three *TDPK* genes (Supplementary Table 2). According to the prediction programs Predotar, TargetP, and PSORT, TDPK1 is targeted to the chloroplast, TDPK2 most likely to the mitochondria, and TDPK3 may be located in the chloroplast or the cytosol. Although these predictions would require experimental proofs, they suggest that rice TDPKs are located in at least two different subcellular compartments. This contrasts with results in *Arabidopsis* that showed both TDPK homologs are located in the cytosol (Ajjawi et al., 2007a). If TDPK1 is required to be localized to the chloroplast, thiamin profiles within chloroplasts may be different in the overexpressing plants than in the control, but the pool of thiamin and its phosphate esters in the chloroplast may represent only a small portion of total cellular thiamin pool. Subcellular localization experiments and thiamin profiling in organelles are warranted to confirm this hypothesis. It will also be interesting to test the effect of crossing TDPK1 lines with THI1 × THIC lines on thiamin level and profile.

Finally, neither thiamin-accumulating THI1 × THIC lines nor TDPK1-overexpressing lines showed enhanced resistance to *X. oryzae* pv. *oryzae* infection based on measurements of lesion lengths. It may be that protein and thiamin levels in THI1 × THIC plants may not be sufficiently high to confer resistance to *X. oryzae* pv. *oryzae*. Priming of plants against pathogens by exogenous application of thiamin required millimolar concentrations of thiamin (Ahn et al., 2005; Bahuguna et al., 2012; Boubakri et al., 2012; Zhou et al., 2013). Further enhancing thiamin content, by crossing THI1 × THIC lines that express high levels of the corresponding proteins with TDPK1 overexpressing lines for instance, may be more effective in enhancing resistance to *X. oryzae* pv. *oryzae*.

## Conclusion

This study shows that overexpression of both THI1 and THIC is the minimal requirement for thiamin accumulation in leaves and seeds of rice. Although engineered seeds accumulated up to fivefold more thiamin than the control, most or all of the increase occurred in the bran. The endosperm thiamin content remained the same as in the control. In addition, thiamin-accumulating THI1 × THIC lines were not resistant to *X.oryzae* pv. *oryzae*, showing that much remains to be understood about how thiamin and/or thiamin metabolism genes relate to disease resistance.

## Author Contributions

WD, NT, PR, and AG conceived and designed the experiments; WD, NT, and AG performed the experiments and analyzed the data; WD, NT, PR, and AG wrote the manuscript.

## Conflict of Interest Statement

The authors declare that the research was conducted in the absence of any commercial or financial relationships that could be construed as a potential conflict of interest.

## Footnotes

1 http://faostat3.fao.org/home/E

2 http://ricearray.org/index.shtml
