# Supplementary material for: Overexpression of Thiamin Biosynthesis Genes in Rice Increases Leaf and Unpolished Grain Thiamin Content But Not Resistance to Xanthomonas oryzae pv. oryzae
Source: Front Plant Sci. 2016 May 10;7:616. doi: 10.3389/fpls.2016.00616 (PMC4861732; doi:10.3389/fpls.2016.00616)
Supplement: Supplementary file 1 [file Data_Sheet_1.DOCX]

# Supporting information

**Supplementary Figure 1.** PCR genotyping of rice plants. (**A)** PCR genotyping of T_0_ plants regenerated on medium containing hygromycin as selective agent. Top panel: PCR genotyping of TDPK1 plants. Bottom panel: Top lane, PCR genotyping of THI1 plants; bottom lane, PCR genotyping of THIC plants. **(B)** PCR genotyping of T_1_ progenies of THI1 and THIC lines. THI1-11 and THI1-12 were regenerated on medium containing mannose as selective agent. THI1-11 was used as negative control in further experiments. W, water control. **(C)** PCR genotyping of T_1_ progenies of TDPK1 lines 2, 19, and 24. **(D)** PCR genotyping of T_2_ progenies of THI1-7-31 and THIC-1-30 lines. All positive plants were used in reciprocal crosses to generate F_1_ hybrids. **(E)** PCR genotyping of F_1_ plants. Lines 1 to 38 were genotyped for *THIC* and *THI1*. Lines 1, 5, 8, 25, and 31 were advanced to F_2_ and F_3_ generations. **(F)** PCR genotyping of F_2_ plants from F_1_ lines 1, 5, 8, 25, and 31. Fifty plants were genotyped for *THIC* and *THI1* and used for *Xoo* resistance tests, and 24 were used for thiamin determination. Primers used for genotyping are shown in Supplementary Table 1.

**A
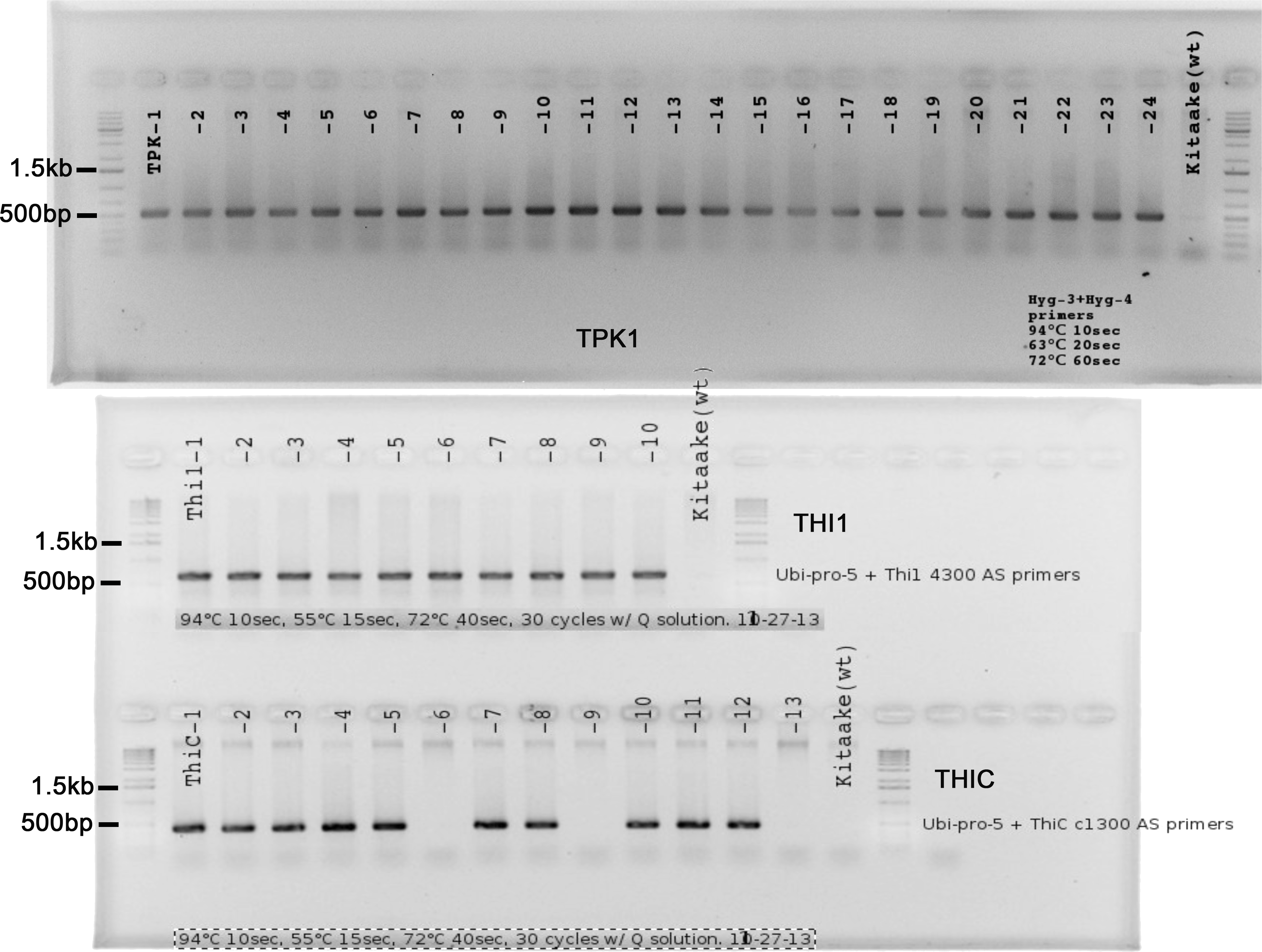
**

**B**


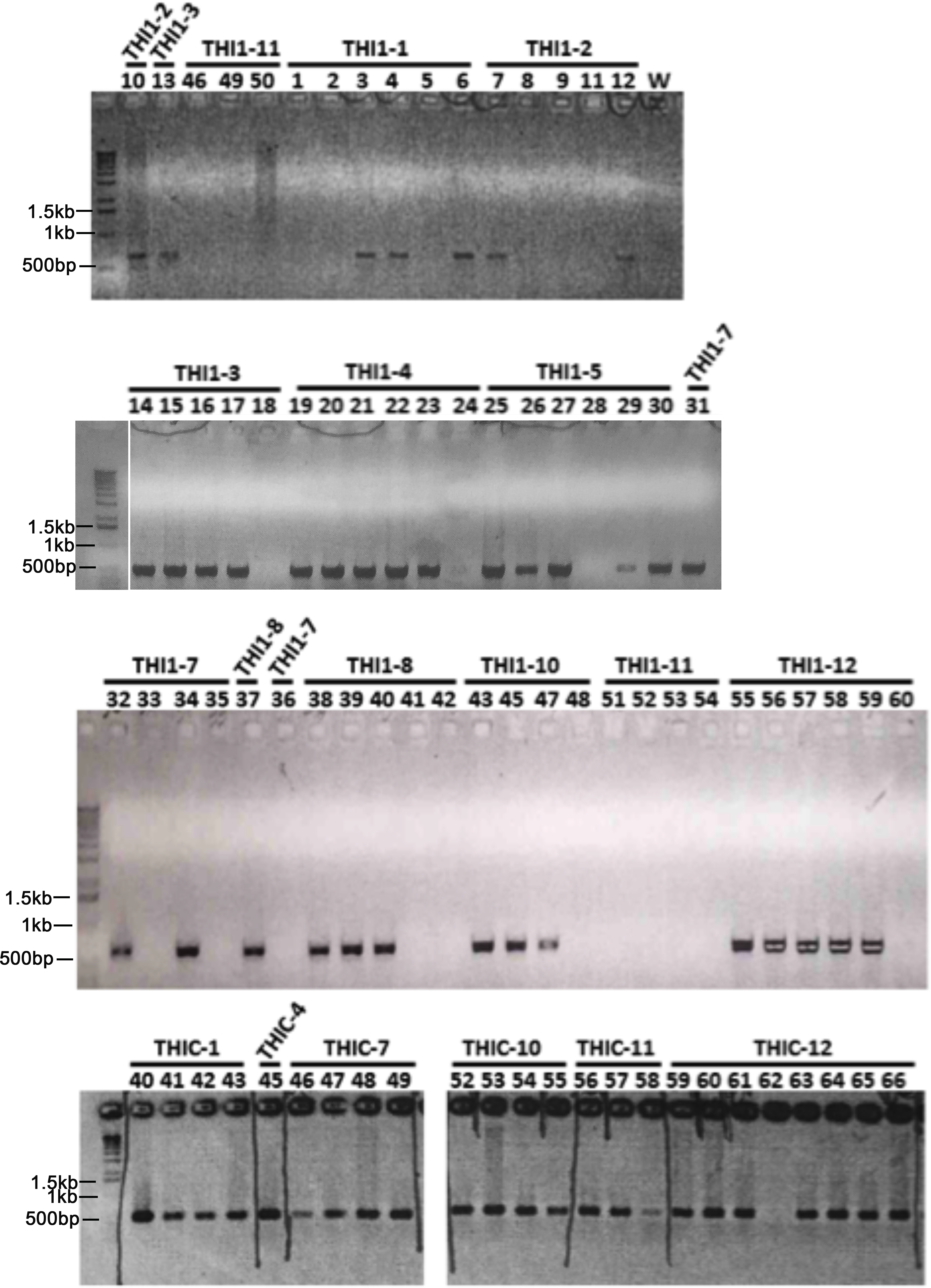


**C**


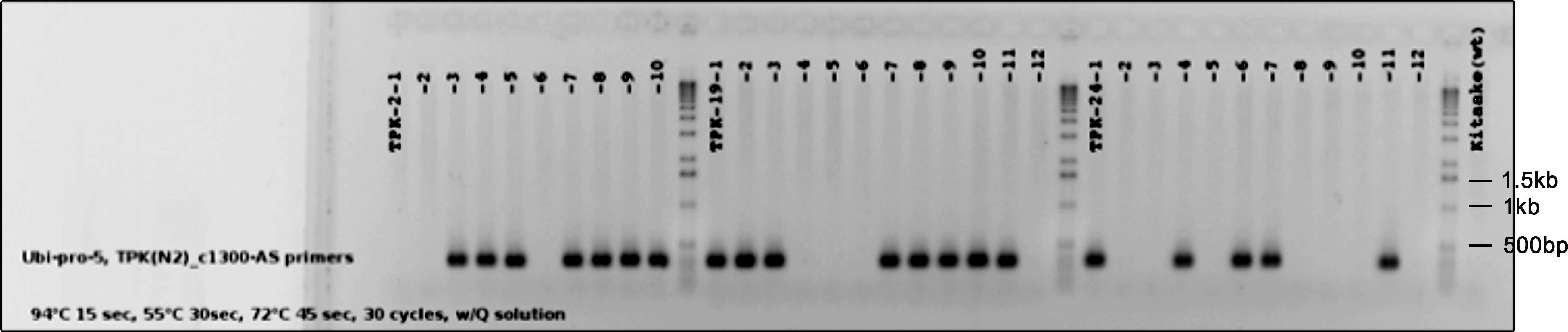


**D**

**
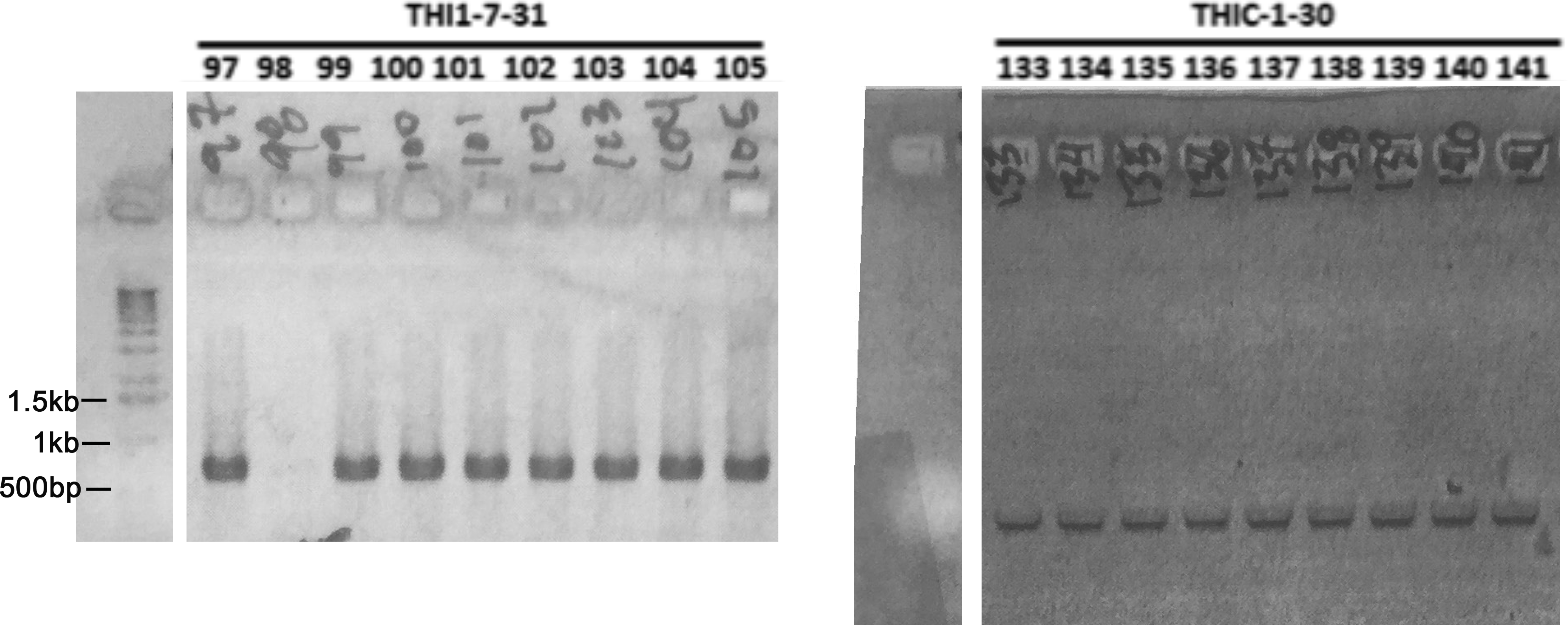
**

**E
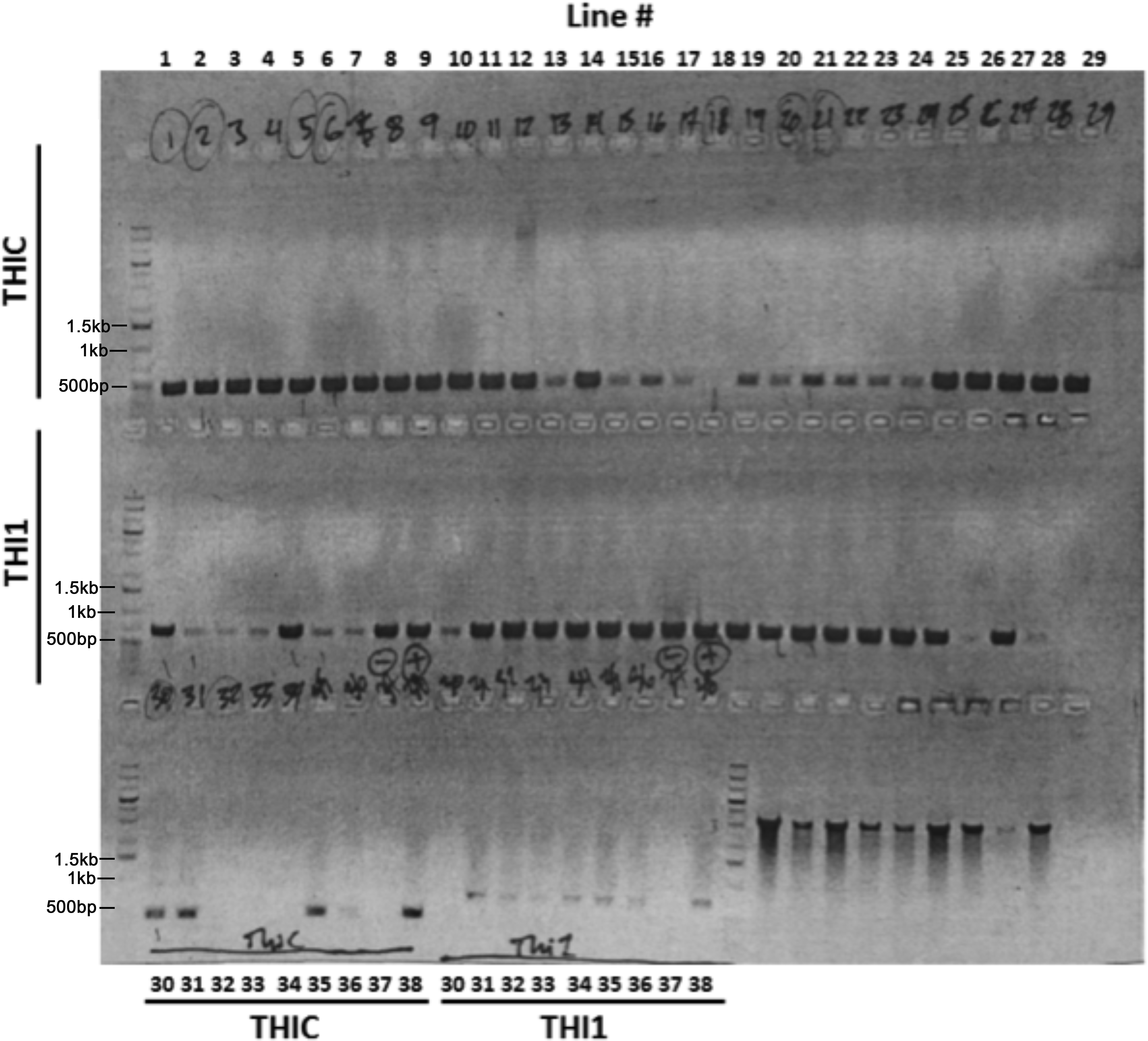
**

**F**
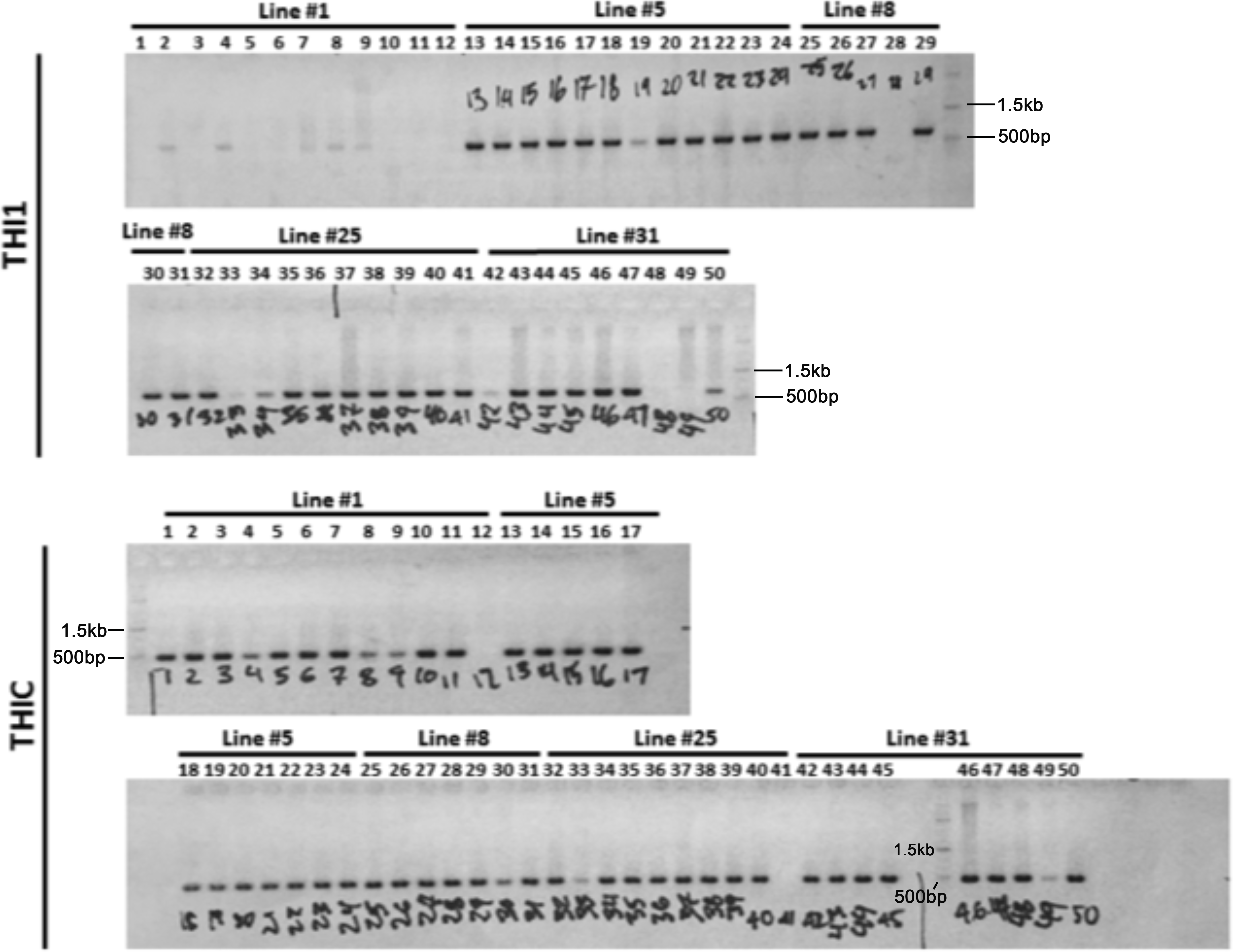


**Supplementary Figure 2.** Evaluation of *Xoo* resistance in T_1_ THI1 (A), T_1_ THIC (B), and T_0_ and T_1_ TDPK1 (C and D) lines. All plants were genotyped by PCR. All T_0_ TDPK1 lines contained the transgenes as shown in Supplementary Figure 1A. Segregating negative and positive T_1_ plants were analyzed whenever possible and are indicated with – (grey bars) or + (black bars), respectively. T_1_ plants used for *Xoo* inoculation were THIC #40 to 66, THI1 #1 to 60, and TPK-2 #1 to 10, TPK-19 #1 to 12, and TPK-24 #1 to 12 (Supplementary Figures 1B and C). Kitaake and Xa21-Kitaake were used as negative and positive controls, respectively. The numbers in parenthesis indicate the number of independent T_1_ plants tested per line. Data are means ± SE. Identical letters indicate that there was no significant difference between samples as determined by ANOVA (*p*>0.05). Note that line THI1-6 was not tested because T_0_ THI1-6 plant did not produce seeds.


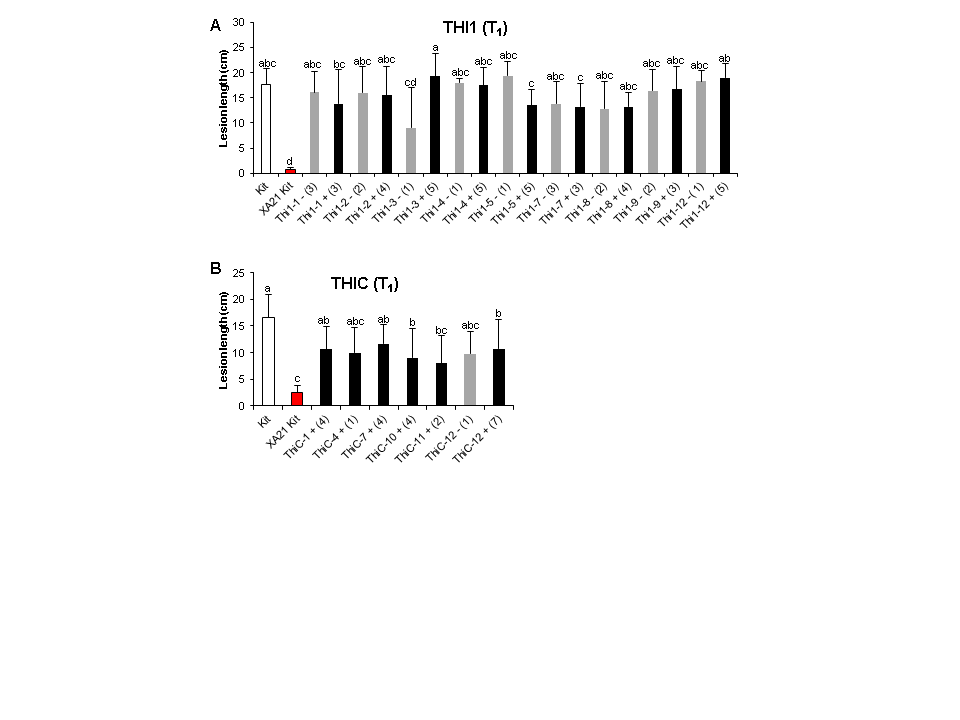


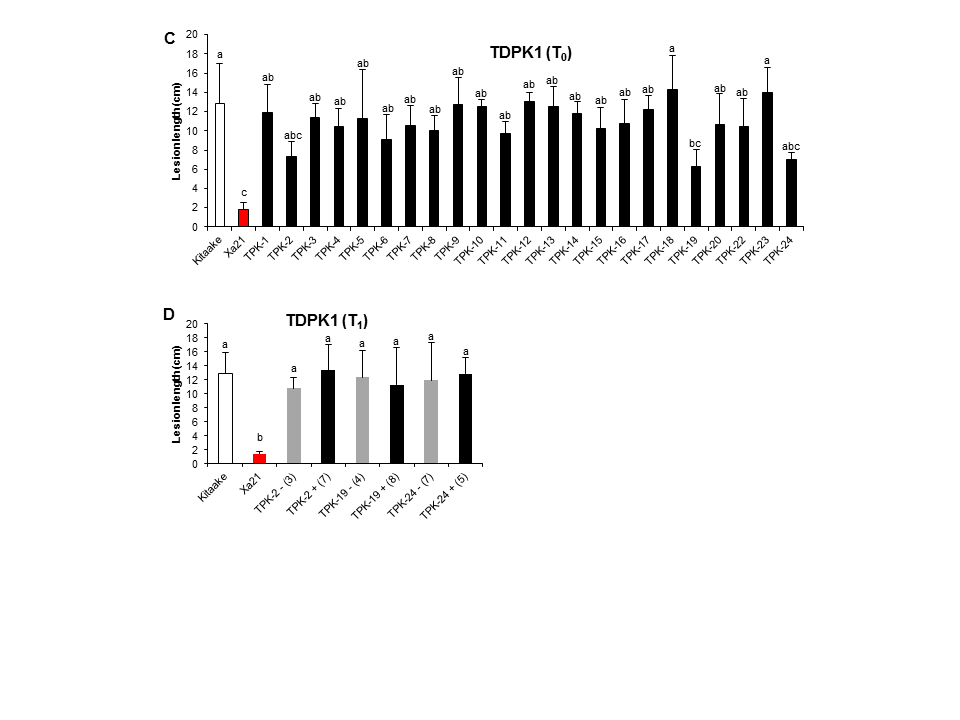


**Supplementary Figure 3.** Correlation between lesion lengths and thiamin (A), ThMP (B), ThDP (C), or total thiamin content (D). F_2_ plants were inoculated with *Xoo* (strain PXO99A). Average lesion lengths were measured 2 weeks after *Xoo* inoculation. Measurements were taken from wild type Kitaake (n = 25) and F_2_ individuals (n = 196). Thiamin analysis was done on 24 F_2_ plants and Kitaake.


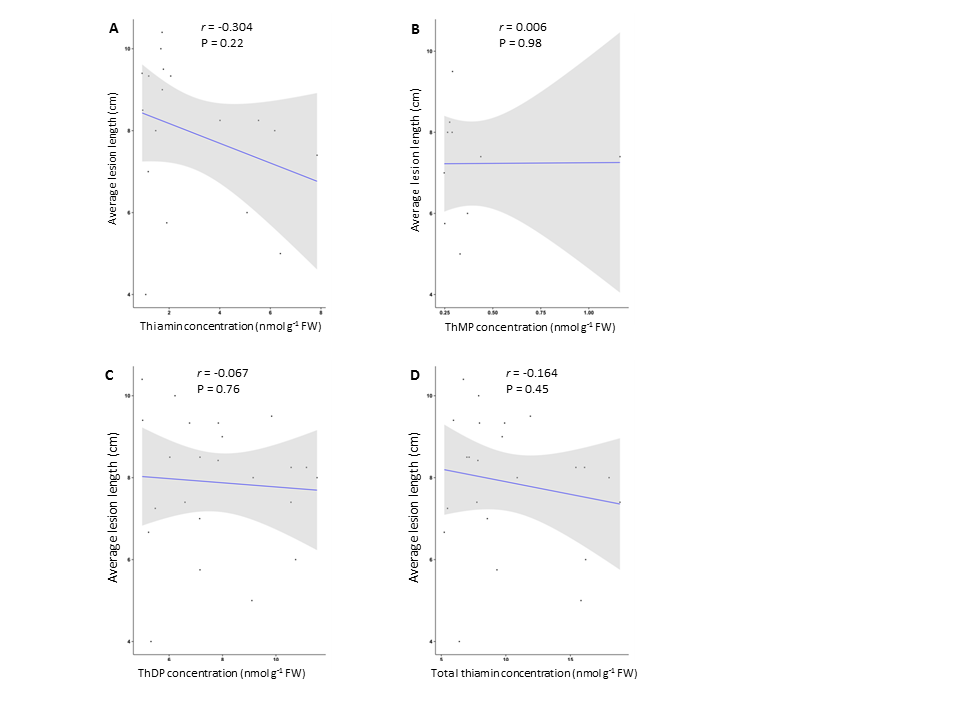


**Supplementary Table 1** Primers used in this study.

| **Primer Name** | **Primer sequence 5’ 🡪 3’** | **Purpose** |
| --- | --- | --- |
| OsThiC-Fwd | CACCGAGGAAATGGCTGCCCT | Cloning of *THIC* |
| OsThiC-Rev | AGACGATCTTATTTGCGAGCTGTA | Cloning of *THIC* |
| OsThi1-Fwd | CACCAGAGCAAGAAGCTCA | Cloning of *THI1* |
| OsThi1-Rev | TGTTCTATTCGCTCAGGCG | Cloning of *THI1* |
| Ubi promoter F | TTGTCGATGCTCACCCTGTTGTTT | Genotyping |
| Thi1_C4300_AS | ATGACGGTGGAGGTGAAGAG | Genotyping |
| ThiC_C1300_AS | GGACATGGCCAGATTCCTC | Genotyping |
| TPK(N2)_C1300_AS | TGTACCTCATGCGGACCTG | Genotyping |
| Ubiquitin forward | GCTCCGTGGCGGTATCAT | Real-time QRTPCR |
| Ubiquitin reverse | CGGCAGTTGACAGCCCTAG | Real-time QRTPCR |
| THI1 forward | CGCCGTCGAGGACCTCAT | Real-time QRTPCR |
| THI1 reverse | GACTGCGTGTCGTGGTTCATC | Real-time QRTPCR |
| THIC forward | GAAGCATTTTGACACAT- ACGAC | Real-time QRTPCR |
| THIC reverse | CACCTAACTTCTCCCTCCTG | Real-time QRTPCR |
| TPK forward | TCCGCATGAGGTACAAGC | Real-time QRTPCR |
| TPK reverse | ATTCATCAACTATTTCGGCAC | Real-time QRTPCR |

#

**Supplementary Table 2** Thiamin metabolism genes of Arabidopsis, maize, and rice.

| **Abbreviation** | **Biochemical**  **function** | **Arabidopsis gene** | **Maize gene** | **Rice gene** | **Rice gene** | **Array Element ID** |
| --- | --- | --- | --- | --- | --- | --- |
| THI1 | Thiazole  biosynthetic protein | TZ, At5g54770 | Thi1, GRMZM2G018375  Thi2, GRMZM2G074097 | Os07g0529600 | LOC_Os07g34570 | Os.12767.1.S1_a_at |
| THIC | Hydroxymethylpyrimidine phosphate synthase | PY, At2g29630 | GRMZM2G027663 | Os03g0679700 | LOC_Os03g47610 | Os.18490.1.S1_x_at (long 3’UTR)  Os.18490.3.S1_at (short 3’UTR) |
| TH1 | Hydroxymethylpyrimidine phosphate kinase/hydroxymethylpyrimidine kinase/thiamin phosphate pyrophosphorylase | At1g22940 | GRMZM2G401934 | Os12g0192500 | LOC_Os12g09000 | Os.12097.1.S1_at |
| ThMPase | Thiamin monophosphate phosphatase | At4g29530 | GRMZM2G035134 | Os01g0720400 | L​O​C​_​O​s​0​1​g​5​2​2​3​0 | Os.12535.1.S1_at |
| TDPK | Thiamin diphosphokinase | TDPK1, At1g02880  TDPK2, At2g44750  TDPK3 | GRMZM2G055458  GRMZM5G864815 | Os01g0931400  Os01g0356500  Os05g0367400 | LOC_Os01g70580  LOC_Os01g25440  LOC_Os05g30454 | Os.52120.1.S1_x_at  Os.34588.1.S1_at  Os.55625.1.S1_at |
| HETK | Hydroxyethylthiazole kinase | At3g24030 | GRMZM2G094558 | Os01g0363600 | PUT-163a-Oryza_sativa_Japonica_Group-130183 (No gene model) | Os.51772.1.S1_at |
| TENA1 | Thiaminase I | At5g32470 | GRMZM2G078283  GRMZM2G148896 | Os08g0566000 | LOC_Os08g45160 | Os.24689.1.S1_at |
| TENA2 | Thiaminase II homolog | At3g16990 | GRMZM2G080501 | Os03g0306900 | LOC_Os03g19390 | Os.11668.1.S1_at |
| NUDIX | Oxy-, oxo-thiamin phosphatase | NUDT20, At5g19460  NUDT24, At5g19470 | GRMZM2G031461 | Os09g0322200 | LOC_Os09g15340 | Os.25015.1.A1_at |
| COG0212 | Thiamin associated 5-formyltetrahydrofolate cycloligase paralog | At1g76730 | GRMZM2G001904 | Os12g0168000 | LOC_Os12g07020 | Os.19306.1.S1_at |
| TPC | Mitochondrial thiamin diphosphate transporter | TPC1, At3g21390  TPC2, At5g48970 | GRMZM2G124911  GRMZM2G118515 | Os01g0934200 | LOC_Os01g70800 | Os.11148.1.S1_at |
